# Contemporary Landscape of Active Clinical Trials in Pancreatic Ductal Adenocarcinoma: A ClinicalTrials.gov—Based Narrative Review with a Scoping Approach

**DOI:** 10.3390/ph19091371

**Published:** 2026-08-30

**Authors:** Laura Radoš, Sara Matulić Čubranić, Marin Golčić, Iva Skočilić, Ivana Mikolašević, Andrej Belančić

**Affiliations:** 1 Tumor Clinic, Clinical Hospital Centre Rijeka, 51000 Rijeka, Croatia; laura1301rados@gmail.com (L.R.); saramatulic@gmail.com (S.M.Č.); iskocilic@gmail.com (I.S.); ivana.mikolasevic@gmail.com (I.M.); 2 Department of Oncology and Radiotherapy, Faculty of Medicine, University of Rijeka, 51000 Rijeka, Croatia; 3 Department of Internal Medicine, Faculty of Medicine, University of Rijeka, 51000 Rijeka, Croatia; 4 Department of Basic and Clinical Pharmacology and Toxicology, Faculty of Medicine, University of Rijeka, 51000 Rijeka, Croatia; a.belancic93@gmail.com

**Keywords:** PDAC, precision oncology, chemotherapy, KRAS inhibition, clinical trials

## Abstract

**Background:** Pancreatic adenocarcinoma (PDAC) remains one of the most lethal malignancies, with limited survival gains despite advances in systemic therapy. However, rapid expansion of biomarker-directed therapies, immunotherapy, and novel treatment modalities has created an increasingly complex clinical trial landscape. We aimed to characterize contemporary PDAC clinical development and identify emerging therapeutic trends. **Methods:** We performed a narrative review with a scoping approach of active PDAC clinical trials registered on ClinicalTrials.gov. Eligible studies were initiated between January 1, 2021, and February 16, 2026, and included recruiting, active, or not-yet-recruiting phase I–III trials. Data extracted included trial phase, disease setting, therapeutic strategy, endpoints, enrollment, sponsorship, and late-phase development. **Results:** A total of 355 interventional trials were included. Most trials were phase I, phase I/II, or phase II trials, with fewer than 10% being evaluated in phase II/III or phase III development. Advanced or metastatic disease was the predominant setting. Chemotherapy remained the most frequently incorporated treatment modality, while molecularly targeted therapies were evaluated in 167 trials. KRAS/RAS-directed approaches represented the largest targeted subgroup, although only daraxonrasib and setidegrasib reached phase III evaluation. Immunotherapy was evaluated in 139 trials, although only a limited number progressed to late-phase development, reflecting the immune-resistant biology of pancreatic adenocarcinoma. Additional areas of active investigation included Claudin 18.2-targeted therapies, MTAP-associated approaches, homologous recombination deficiency-directed strategies, CD73 inhibition, radiotherapy, local interventions, surgery-focused optimization, imaging-guided approaches, and supportive-care interventions. Industry organizations were listed as the lead sponsor for approximately half of all studies, while non-commercial organizations supported most remaining trials. **Conclusions:** The contemporary PDAC clinical trial landscape is characterized by broad therapeutic diversification but limited late-phase maturity. Chemotherapy remains the dominant treatment backbone, whereas KRAS/RAS-directed therapies have emerged as the most advanced precision oncology strategy. Future progress will likely depend on successful integration of biomarker-selected therapies with established multidisciplinary treatment approaches, including optimized systemic therapy, local-control strategies, and supportive care.

## 1. Introduction

PDAC remains one of the most lethal malignancies worldwide and is projected to become an increasingly prominent cause of cancer-related mortality over the coming decades. Despite representing a relatively small proportion of all cancer diagnoses, PDAC accounts for a disproportionate burden of cancer deaths owing to its aggressive biology, late presentation, and limited responsiveness to available therapies [1]. Most patients are diagnosed with unresectable locally advanced or metastatic disease, and even among those who undergo potentially curative surgery, recurrence rates remain high. Combination chemotherapy regimens such as FOLFIRINOX and gemcitabine plus nab-paclitaxel have become established standards of care in advanced disease, while perioperative treatment approaches have expanded the role of multimodal management in localized disease [2,3]. However, long-term survival remains poor, with contemporary population-based estimates reporting a 5-year overall survival of only approximately 13% [2,3,4,5].

Recent advances in tumor genomics, molecular profiling, and translational cancer biology have fundamentally reshaped the PDAC research landscape. Increasing recognition of molecular heterogeneity has facilitated the development of biomarker-directed therapeutic strategies targeting specific genomic alterations, including KRAS mutations and homologous recombination deficiency-associated pathways [6,7]. Simultaneously, a broad spectrum of innovative treatment modalities has entered clinical evaluation, including antibody-drug conjugates, cellular therapies, therapeutic vaccines, immune-modulating agents, stromal-targeting approaches, radiotheranostic platforms, and novel local-regional interventions [8].

Despite the expansion in therapeutic innovation, relatively few investigational approaches ultimately advance to late-stage clinical development or become incorporated into routine clinical practice. Previous analyses of PDAC research have highlighted the predominance of early-phase studies and the substantial attrition that characterizes oncology drug development [9,10]. However, the accelerating diversification of therapeutic strategies and emergence of precision oncology approaches are creating a need for updated assessments of ongoing clinical development. Thus, the aim of this narrative review was to systematically characterize the contemporary PDAC clinical trial landscape. Specifically, we sought to evaluate patterns in trial design, disease setting, therapeutic strategies, endpoint selection, and funding sources, while identifying emerging trends and areas in which ongoing clinical development may be insufficiently aligned with the unmet clinical needs of patients with PDAC.

## 2. Methods

We conducted a comprehensive search of ClinicalTrials.gov [11], the largest international registry of clinical trials, to identify investigational therapies for PDAC. ClinicalTrials.gov lists more than 300,000 studies worldwide—approximately ten times the number registered in the European Union Clinical Trials Register [12]—and was therefore selected as the primary data source given the global scope of contemporary multicenter trials evaluating novel therapeutics, particularly those seeking regulatory approval in the United States, Europe, and other jurisdictions.

On 16 February 2026, two independent reviewers (A.B. and M.G.) systematically searched ClinicalTrials.gov using predefined criteria: indication (pancreatic ductal adenocarcinoma, including “PDAC”, “Pancreatic adenocarcinoma”, and “Pancreas Adenocarcinoma”); recruitment status (not yet recruiting, recruiting, or active but not recruiting); age group (adult [18–64 years] and older adult [≥65 years]); study phase (early phase I, phase I, phase I/II, phase II, phase II/III or phase III); study type (interventional); and study start date (on or after 1 January 2021). Subsequently published results available up to June 2026 were used only to contextualize selected late-phase programs.

Eligible studies were restricted to interventional trials evaluating investigational therapeutic, diagnostic-guided, local-control, supportive-care, or multimodality strategies relevant to PDAC management. Novelty was defined as use of agents not approved for PDAC, new combinations, alternative dosing or delivery approaches, device-based interventions, or novel integration of established modalities within a trial-defined treatment strategy.

Completed, terminated, withdrawn, and suspended trials were excluded because the objective was to characterize the currently active investigational pipeline rather than all trials initiated during the study period.

An approximately 5-year capture window was selected to allow sufficient time for identification and developmental progression of candidate therapies that might advance towards regulatory review, consistent with typical timelines for interim analyses and decision-making in active trials. Moreover, this timeframe was selected to capture the contemporary post-standard-of-care treatment landscape, including the development of therapies beyond FOLFIRINOX and gemcitabine plus nab-paclitaxel and the increasing emergence of biomarker-driven clinical trials.

Data were extracted into a prespecified Microsoft Excel database and included NCT number and other identifiers, study title and URL, phase, geographic location, investigational intervention, eligibility criteria, study outcomes, start and completion dates, anticipated or actual enrolment, recruitment status, sponsor, and collaborators.

## 3. Results

### 3.1. Trial Characteristics

A total of 355 PDAC clinical trials met the eligibility criteria and were included in the analysis [Supplementary Table S1. Note for Supplementary Table S1: Clinical trials classified as phase II/III or phase III are highlighted in yellow in the “Phase” column. A yellow cell indicates that the trial meets these phase criteria]. At the time of data extraction, 232 trials (65.3%) were recruiting, 89 (25.1%) were active but not recruiting, and 34 (9.6%) had not yet initiated recruitment.

Phase I trials represented the largest proportion of trials (*n* = 110, 31.0%), followed by phase II (*n* = 109, 30.7%) and phase I/II trials (*n* = 90, 25.4%). Early phase I trials accounted for 15 trials (4.2%). Only 31 trials (8.7%) had progressed to late-phase clinical development, comprising 11 phase II/III (3.1%) and 20 phase III (5.6%) trials.

Planned enrollment ranged from 3 to 5000 participants, with a median enrollment of 68 patients (95% CI 55.2–82.2). Planned trial duration ranged from 6.1 to 235.9 months, with a median duration of 46.4 months (95% CI 42.8–47.9 months).

Allocation was most frequently reported as not applicable (*n* = 153, 43.1%), followed by non-randomized design (*n* = 124, 34.9%), while 78 trials (22.0%) were randomized. According to intervention models, single-group designs were the most common (*n* = 146, 41.1%), followed by parallel-group (*n* = 106, 29.9%) and sequential designs (*n* = 102, 28.7%), while only one trial used factorial design (0.3%).

Most trials were open label (*n* = 337, 94.9%), with a small proportion incorporating any form of blinding (*n* = 18, 5.1%), with quadruple masking in 7 trials (2.0%), single masking in 5 (1.4%), triple masking in 2 (0.6%), and double masking in 4 (1.1%).

Using non-mutually exclusive disease-stage categories, 243 trials (68.5%) included patients with advanced or metastatic disease, 71 (20.0%) included locally advanced disease, 20 (5.6%) included borderline resectable disease, and 32 (9.0%) included resectable disease. Refractory and recurrent disease were each represented in 18 trials (5.1%). Twenty-four trials (6.8%) evaluated neoadjuvant approaches, and eight (2.3%) evaluated adjuvant approaches. Disease stage was not clearly specified in 40 trials (11.3%). Because individual trials could included mixed-stage populations, these categories were not mutually exclusive.

#### 3.1.1. Endpoint Characteristics

**Figure 1:**
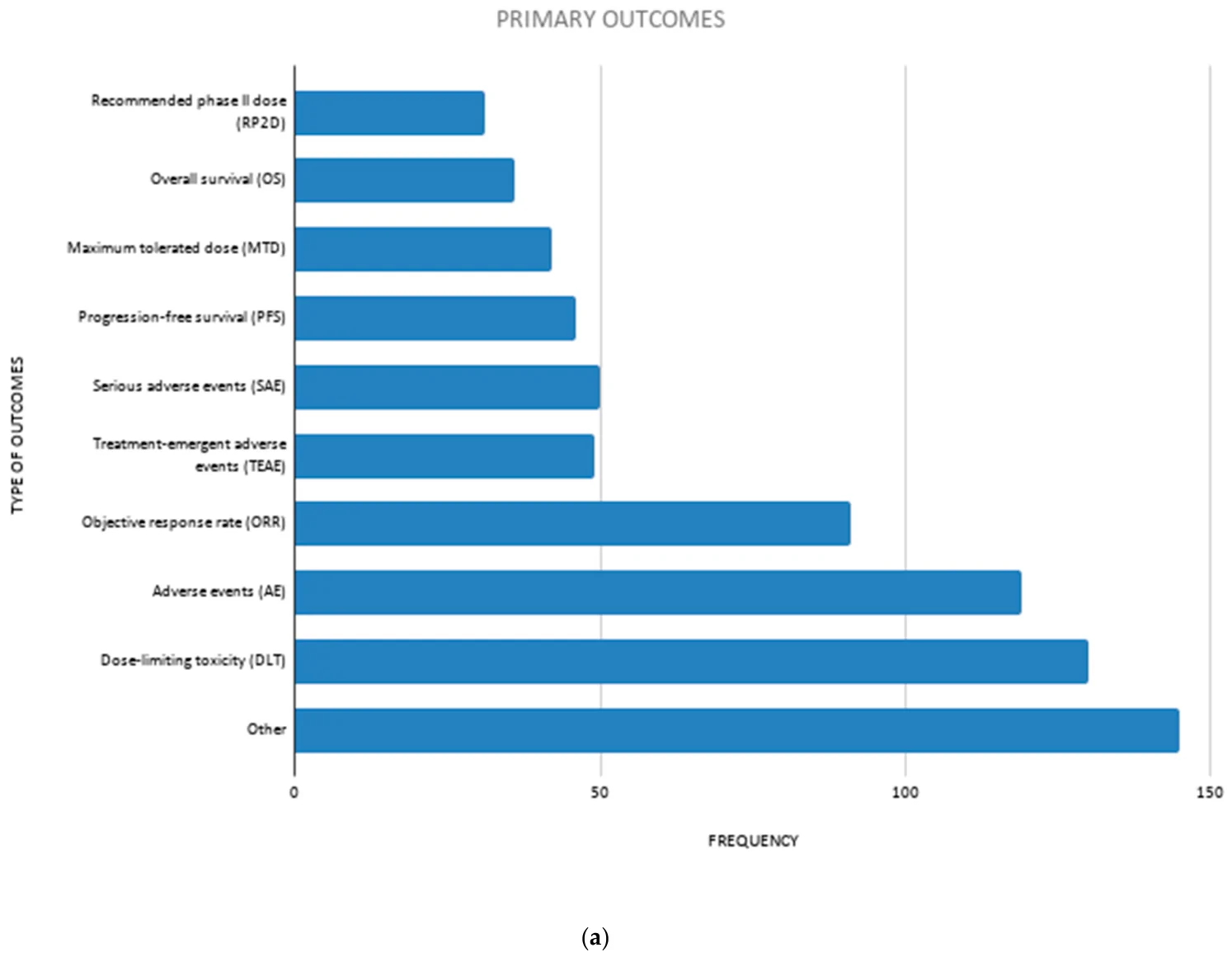

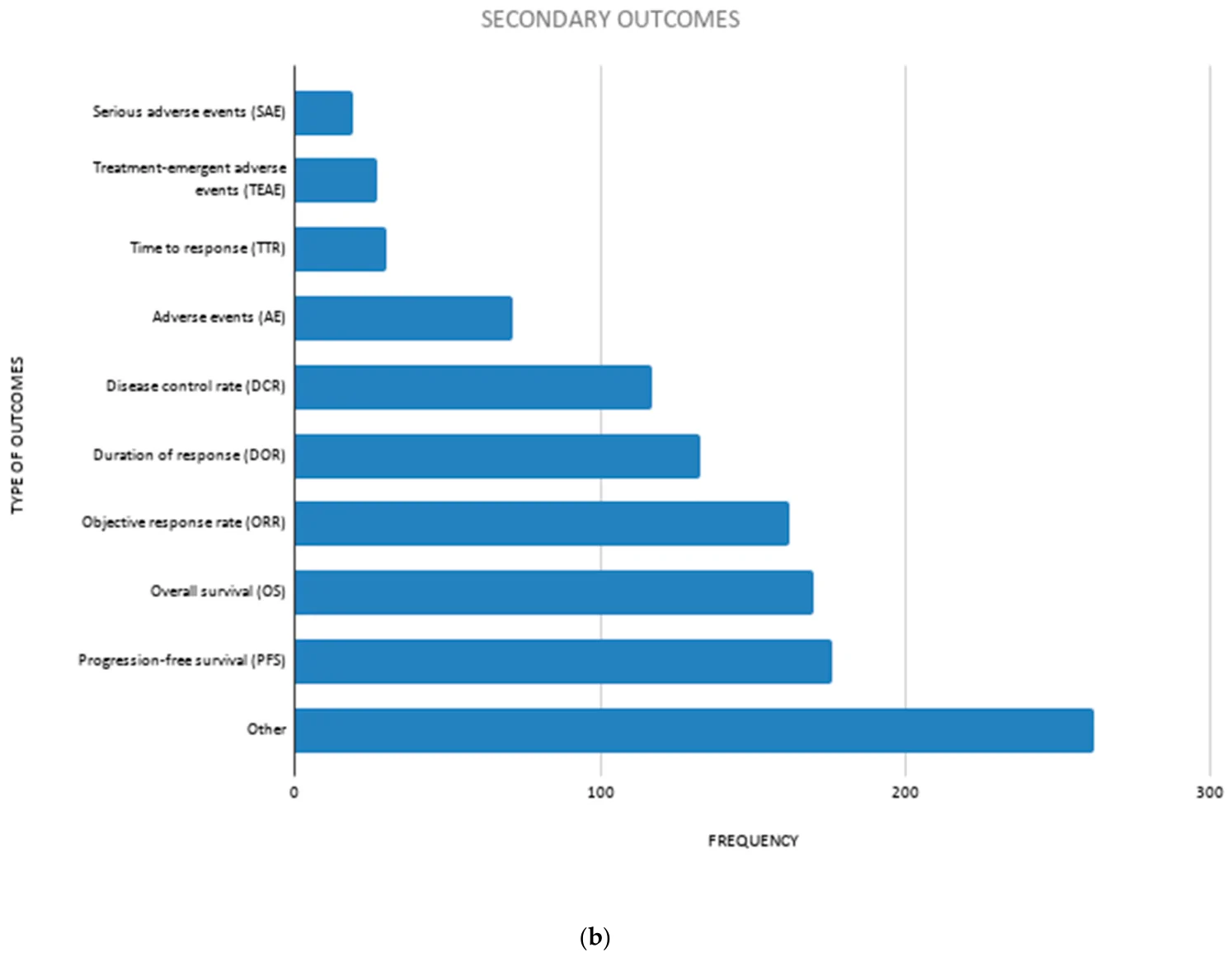
(**a**). Most common primary endpoints in ongoing clinical trials for PDAC patients. Trials could have more than one primary endpoint. (**b**). Most common secondary endpoints in ongoing clinical trials for PDAC patients. Trials could have more than one secondary endpoint. Note: X-axis: Frequency, defined as the absolute number of trials reporting each outcome.

Primary endpoint measures most commonly included other outcomes (*n* = 145, 40.8%), followed by dose-limiting toxicity (DLT) (*n* = 130, 36.6%), adverse events (AE) (*n* = 119, 33.5%), and objective response rate (ORR) (*n* = 91, 25.6%). Additional frequently reported primary endpoints included serious adverse events (SAE) (*n* = 50, 14.1%), treatment-emergent adverse events (TEAE) (*n* = 49, 13.8%), progression-free survival (PFS) (*n* = 46, 13.0%), maximum tolerated dose (MTD) (*n* = 42, 11.8%), overall survival (OS) (*n* = 36, 10.1%), and recommended phase II dose (RP2D) (*n* = 31, 8.7%) (Figure 1a). Trials could report more than one primary outcome measure. The “Other” primary endpoint category (*n* = 145, 40.8%) included pharmacokinetic and pharmacodynamic measures, biomarker and translational endpoints, imaging outcomes, feasibility assessments, and procedure-specific technical outcomes that did not fit predefined efficacy or safety categories.

There were substantially more reported secondary outcomes. The most common secondary outcome category was other outcomes (*n* = 262, 73.8%), followed by progression-free survival (PFS) (*n* = 176, 49.6%), overall survival (OS) (*n* = 170, 47.9%), objective response rate (ORR) (*n* = 162, 45.6%), duration of response (DoR) (*n* = 133, 37.5%), and disease control rate (DCR) (*n* = 117, 33.0%). Additional secondary endpoints included adverse events (AE) (*n* = 71, 20.0%), time to response (TTR) (*n* = 30, 8.5%), treatment-emergent adverse events (TEAE) (*n* = 27, 7.6%), and serious adverse events (SAE) (*n* = 19, 5.4%) (Figure 1b). As studies frequently reported multiple secondary outcome measures, the total number of reported outcomes exceeded the total number of included studies. The “Other” secondary endpoint category (*n* = 262, 73.8%) included categories previously reported for primary outcomes.

#### 3.1.2. Trial Sponsors

Industry was the most common lead sponsor for funding, supporting 49.3% (*n* = 175) of included studies, while “other” funding sources accounted for 47.3% (*n* = 168). Only a small proportion of studies were funded by the National Institutes of Health (NIH) (*n* = 8, 2.3%) or research networks (*n* = 4, 1.1%).

### 3.2. Treatment Landscape

Treatment modalities were analyzed separately by therapeutic category. However, categories were not mutually exclusive because many trials evaluated combination strategies involving chemotherapy, targeted therapy, immunotherapy, radiotherapy, surgery or local interventions. Of the 355 trials, 234 (65.9%) evaluated combinations of at least two agents or interventions, whereas 121 (34.1%) tested a single agent or intervention.

#### 3.2.1. Chemotherapy

Chemotherapy remained the most frequently incorporated treatment modality and was present in 171 trials (48.2%). A total of 19 (5.4%) evaluated chemotherapy alone, while the rest (*n* = 152, 42.8%) evaluated combined therapeutic approaches. Among chemotherapy-containing trials, 112 (31.5%) combined chemotherapy with other systemic therapies, and 40 (11.3%) combined chemotherapy with local therapies, radiotherapy, and/or surgery, with or without an additional systemic agent. Overall, 20 chemotherapy-containing trials (11.7% of chemotherapy-based trials) were evaluated in late-phase including 13 (7.6% of chemotherapy-based trials) in phase III.

#### 3.2.2. Targeted Therapy

Molecularly targeted therapies were evaluated in 167 trials (47.0%). Targeted therapies were classified according to their primary molecular target regardless of the type of molecule.

Kirsten Rat Sarcoma/Rat Sarcoma (KRAS/RAS)-directed approaches constituted the largest targeted subgroup (*n* = 42, 11.8%). This category consisted of a diverse range of therapeutic strategies, including direct KRAS/RAS inhibitors and degraders, mutation-specific agents, pan-RAS inhibitors, KRAS-targeted vaccines, adoptive cellular therapies, and KRAS G12D siRNA-based approaches. KRAS G12D was the most frequently specified mutation in this group. Daraxonrasib was the most commonly investigated KRAS-directed agent, which reached phase III evaluation in two trials (NCT07252232, NCT06625320), while setidegrasib, a KRAS G12D degrader, was evaluated in one phase III trial, combined with chemotherapy (NCT07409272).

Claudin 18.2-directed approaches represented the second most-common targeted approach (*n* = 18 trials, 5.1%). This included monoclonal antibodies, antibody-drug conjugates, chimeric antigen receptor (CAR) T cell therapies, T-cell engagers, and bispecific antibodies. However, all Claudin 18.2-directed studies remained in advanced or mixed settings with early-phase trials only and no late phase trials identified.

Methylthioadenosine phosphorylase (MTAP) deletion as a target was evaluated in 9 trials (2.5%). This subgroup primarily consisted of protein arginine methyltransferase 5 (PRMT5) and methionine adenosyltransferase 2A (MAT2A) inhibitors. One MTAP-deletion associated agent, BMS-986504 (a PRMT5 inhibitor) reached phase II/III assessment combined with chemotherapy in advanced disease settings (NCT07076121).

Homologous recombination deficiency (HRD)-associated approaches were evaluated in 9 trials (2.5%). Most studies investigated poly-ADP-ribose polymerase (PARP) inhibitors, including olaparib, niraparib, and EIK1004, and/or evaluated patients with breast cancer gene 1 or 2 (BRCA1/2), partner and localizer of BRCA 2 (PALB2), or other HRD-associated alterations. Three HRD-associated trials progressed to phase II/III or III development; however, all of them evaluated platinum-based treatment strategies in molecularly selected HRD populations (NCT06783140, NCT06115499, NCT06095141).

Mesothelin expression was a target in 7 trials (2.0%), predominantly involving cell-based therapy and targeted vaccines, but also included T-cell engagers and antibodies. All trials were conducted in early-phase settings and were primarily evaluated in advanced and mixed disease settings.

Epidermal growth factor receptor (EGFR)-targeted approaches were identified in 14 trials (3.9%), consisting of monoclonal antibodies, antibody-drug conjugates, or bispecific antibodies, mostly combined with other systemic agents. One EGFR-targeted trial progressed to phase III development, evaluating panitumumab in combination with chemotherapy in mixed-stage patients with KRAS wild-type PDAC (NCT06998940).

Cluster of differentiation 73 (CD73)-targeted approaches were evaluated in 4 trials (1.1%) and exclusively combined with other systemic agents. Quemlicustat combined with chemotherapy in an advanced-disease setting was evaluated in a phase III trial (NCT06608927).

Other targeted approaches included heterogeneous targeted therapies such as agents directed against focal adhesion kinase (FAK) and mitogen-activated protein kinase signaling (MAPK) pathways, vascular endothelial growth factor (VEGF) and fibroblast growth factor receptor (FGFR) signaling, carcinoembryonic antigen-related cell adhesion molecule 5 (CEACAM5), B7 homolog 3 (B7-H3), trophoblast cell-surface antigen 2 (TROP2), cadherin-17 (CDH17), and several other emerging molecular targets. These approaches were almost exclusively investigated in early-phase clinical development.

#### 3.2.3. Immunotherapy

Immunotherapy was evaluated in 139 trials (39.2%). Checkpoint inhibitors represented the largest subgroup (*n* = 96, 27.0%), followed by a heterogeneous group of immune-modulating therapies (*n* = 32, 9.0%). Cell-based therapies accounted for 26 trials (7.3%) and comprised adoptive cell therapy, including chimeric antigen receptor T-cell (CAR-T), chimeric antigen receptor natural killer cell (CAR-NK), tumor-infiltrating lymphocyte (TIL), and T-cell receptor-engineered T-cell (TCR-T) therapies. Vaccines (*n* = 12, 3.4%) included KRAS-targeted vaccines, mRNA-based vaccines, personalized neoantigen vaccines and other tumor-specific strategies. Oncolytic viruses (*n* = 5, 1.4%) included viruses designed to stimulate antitumor immunity through selective tumor infection and immune activation, while T-cell engagers (*n* = 5, 1.4%) were agents redirecting T-cell activity towards tumor-associated antigens such as Claudin 18.2, EGFR, and CDH17. Most immunotherapeutic strategies remained in early-phase development. Among checkpoint inhibitor-containing approaches, two phase III trials were identified, with serplulimab combined with stereotactic body radiation therapy (SBRT) (NCT07336953) and chemotherapy and atezolizumab combined with bevacizumab in circulating DNA (ctDNA)-positive cancers (NCT05482516). Out of immune-modulating therapies, an oligodeoxynucleotide targeting transforming growth factor beta 2 messenger RNA (TGF-β2 mRNA), OT-101, reached phase II/III combined with chemotherapy (NCT06079346). No vaccine-, oncolytic virus-, or T-cell engager-based strategies were represented in late-phase development, while one cell-based agent, Immuncell-LC was identified in a phase III trial combined with chemotherapy (NCT04969731). Across all immunotherapeutic subgroups, combinations of different strategies predominated, with relatively few studies evaluating immunotherapy as a standalone treatment modality.

#### 3.2.4. Radiotherapy, Local Interventions and Surgery

Radiotherapy, local intervention, and surgery-based treatment strategies were identified in 55 trials (15.5% of all included trials).

Radiotherapy-based approaches were evaluated in 28 trials (7.9%). These included SBRT, MRI-guided adaptive radiotherapy, intensity-modulated radiation therapy (IMRT), dose-escalated radiotherapy, metastasis-directed consolidative radiation, brachytherapy, and other advanced radiation techniques. SBRT represented the most frequently investigated radiotherapy platform and was commonly evaluated in combination with chemotherapy and immunotherapy. Four radiotherapy-based trials advanced to phase III evaluation, including trials investigating SBRT combined with serplulimab and chemotherapy (NCT07336953), dose-escalated radiotherapy (NCT06958328), metastasis-directed consolidative radiation (NCT06593431), and IMRT (NCT06250972).

Local interventions accounted for 21 trials (5.9%) and included irreversible electroporation, cryoablation, sonoporation, high-intensity focused ultrasound, radiofrequency ablation, tumor-treating fields, acoustic cluster therapy, intraperitoneal treatment approaches, and other locoregional techniques. Most were investigated in combination with systemic therapy and remained in early-phase development. The only local intervention to reach phase III evaluation was the NanoKnife system, assessed in combination with chemotherapy (NCT03899636).

Surgery-focused strategies represented 7 trials (2.0%) and included perioperative management strategies, reconstruction procedures, fluorescence-guided surgery, and total pancreatectomy with intraportal islet autotransplantation. Three surgery-related phase III trials were identified: one evaluating pancreaticojejunostomy reconstruction techniques (NCT07155525) and two investigating the optimal integration of surgery with systemic therapy in perioperative treatment strategies (NCT07081360, NCT04927780).

#### 3.2.5. Imaging and Supportive Care

Imaging studies were included when they were embedded within treatment selection, radiotheranostic development, or intervention-guidance strategies.

Imaging and theranostic strategies were investigated in 17 trials (4.8%), including advanced imaging-guided treatment approaches, molecular imaging techniques, and radiotheranostic platforms. Two positron emission tomography (PET)-based radiotracers, GEH300079 (68Ga) and [18F]AlF-FAPI-74 were evaluated in phase II/III and none in phase III (NCT07219238, NCT06782412).

Supportive care approaches were evaluated in 11 trials (3.1%). These included care delivery models, comprehensive geriatric assessment, prehabilitation, pancreatic enzyme replacement, metabolic or diabetes-directed interventions, anti-cachexia strategies, thromboembolism prevention, and treatment of chemotherapy-related anemia. Compared with most investigational therapeutic categories, supportive care trials more frequently reached later stages of development, with two phase II/III trials and two phase III trials identified. Phase III trials evaluated kinisoquin for thromboembolic events (NCT06861088) and ascorbate for chemotherapy-related anemia (NCT06018883).

## 4. Discussion

This narrative review with a scoping approach provides a comprehensive overview of active PDAC clinical trials registered between 2021 and 2026, highlighting the rapidly evolving and heterogeneous therapeutic landscape. Unlike previous analyses, which have primarily focused on overall trial activity, therapeutic categories and emerging treatment strategies, our analysis provides an updated overview of the active development pipeline while extending beyond simple categorization of investigational therapies. We examined disease-stage distribution, endpoint selection, funding patterns, and the characteristics of late-phase clinical development (phase II/III and phase III). Moreover, our analysis includes therapeutic approaches that were either not represented or were minimally represented in earlier assessments, such as KRAS-directed therapies, Claudin 18.2-targeted therapies, MTAP-associated strategies, CD73 inhibition, and contemporary biomarker-selected treatment approaches [13,14,15]. Furthermore, we analyzed not only which therapeutic strategies are currently being explored but also which strategies show potential to meaningfully influence future clinical practice.

### 4.1. Trial Characteristics, Disease Settings, and Endpoint Selection

Although 355 interventional trials were identified, the majority remained in early clinical development, with fewer than 10% progressing to phase II/III or phase III evaluation. Together with the relatively small median planned enrolment (*n* = 68) and an overwhelming predominance of open label trials (>90%), these findings illustrate a research landscape dominated by exploratory drug development and with only a small proportion of investigational therapies advancing to late-stage clinical testing. This likely reflects the biological complexity of pancreatic adenocarcinoma, the historically high failure rate of novel agents, and the difficulty of demonstrating clinically meaningful survival benefits in this disease.

The predominance of advanced and metastatic disease in trials (68.5%) reflects the clinical reality of PDAC, where most patients present with unresectable disease and survival remains poor despite incremental therapeutic advances [1,2,3,8]. The growing number of perioperative studies further suggests that innovation in the resectable disease setting is increasingly focused on optimizing treatment sequencing and integration rather than identifying entirely new therapeutic agents.

Approximately half of all included trials were industry sponsored, with a similarly large proportion supported by academic institutions and other non-commercial organizations. This aligns with a previous analysis by Mederos and Girgis, who observed a substantial increase in industry-sponsored PDAC clinical trials between 2003 and 2022, whereas the proportion of federally funded trials remained relatively stable over time [16]. While industry sponsorship is essential for the development of novel agents, it has also been associated with a higher likelihood of favorable study outcomes [17]. This underscores the importance of non-commercial and academically funded research, which is essential to complement industry-led efforts, providing independent evidence and addressing relevant questions beyond experimental drug development.

Adverse events and dose-limiting toxicities were among the most common primary outcomes, whereas PFS, OS, DoR, and DCR were more frequently assessed as secondary endpoints. This is consistent to the fact that most included trials were conducted in early-phase settings, where the main goal is to establish whether the investigated strategy or agent can be applied safely and to determine the optimal dose for later-stage confirmatory trials. The lack of survival-based primary endpoints therefore highlights the need for more late-phase confirmatory trials which can verify whether there is a clinically meaningful benefit of a treatment strategy.

### 4.2. Current Treatment and Chemotherapy

Despite recent therapeutic advances, PDAC remains one of the deadliest solid malignancies, with a reported 5-year relative survival of approximately 13.7% as of early 2026 [8]. Although several novel therapeutic strategies have emerged over the past decade, chemotherapy remains the only treatment modality that has consistently demonstrated meaningful survival benefit across both metastatic and resectable disease. Consequently, current ESMO and NCCN guidelines continue to recommend chemotherapy-based regimens as the backbone of PDAC treatment, while molecular profiling is primarily used to identify the relatively small subset of patients eligible for biomarker-directed therapies [2,3].

In metastatic disease, FOLFIRINOX and gemcitabine plus nab-paclitaxel established the principal first-line standards, while the NAPOLI-3 trial further refined chemotherapy-based management by demonstrating superior survival with NALIRIFOX compared with gemcitabine plus nab-paclitaxel, with a median OS of 11.1 months in the experimental arm [18,19,20]. Similarly, in resectable disease, PRODIGE 24 established modified FOLFIRINOX as the preferred adjuvant regimen, with durable long-term benefit confirmed on extended follow-up, achieving a median overall survival of 53.5 months, although several different chemotherapy options remain for patients unable to tolerate FOLFIRINOX [21,22].

However, our findings demonstrate that the treatment paradigm is changing. Chemotherapy was incorporated into fewer than half of all identified trials (48.2%), although it was included in 13 of the 20 phase III studies. However, only three phase III trials evaluate chemotherapy alone without the addition of other therapeutic modalities, primarily focusing on optimizing delivery rather than evaluating new chemotherapeutic agents. The PANThEON trial (NCT06897644) evaluates maintenance gemcitabine plus nab-paclitaxel following induction-modified FOLFIRINOX in patients with advanced pancreatic adenocarcinoma [23]. Building on the de-escalation strategy explored in PANOPTIMOX-PRODIGE 35, this trial aims to determine whether maintenance chemotherapy can preserve disease control while reducing cumulative toxicity associated with prolonged intensive treatment [24]. In resectable disease, phase III trial NCT06714604 investigates standard versus prolonged neoadjuvant chemotherapy in patients with borderline resectable and locally advanced PDAC, addressing the unresolved question of whether extending preoperative systemic therapy improves tumor downstaging, resection rates, and long-term survival in patients receiving neoadjuvant treatment [25]. Similarly, NCT06571461 compares postoperative liposomal irinotecan, oxaliplatin, and S-1 with gemcitabine plus capecitabine following curative resection [26]. The trial builds upon the evolving evidence supporting intensified perioperative chemotherapy, including the survival benefit demonstrated with modified FOLFIRINOX in PRODIGE 24 and the long-term outcomes of ESPAC-4, while also incorporating liposomal irinotecan, which has shown clinical efficacy in advanced PDAC through the NAPOLI program [21,27,28,29].

### 4.3. Precision Oncology in Pancreatic Adenocarcinoma

One of the most prominent trends identified in our analysis was the rapid expansion of biomarker-driven therapeutic development, reflecting the ongoing transition towards precision oncology, with an increasing proportion of investigational therapies designed to target genetic alterations or biological vulnerabilities within pancreatic adenocarcinoma. However, only a limited number were evaluated in phase III clinical development.

Among all targeted approaches, KRAS/RAS-directed therapy represented the most important finding in our analysis. This is biologically expected because activating KRAS mutations are present in more than 90% of pancreatic ductal adenocarcinomas and represent the dominant oncogenic driver of this disease [6,30,31]. Consequently, KRAS-directed therapies have the potential to benefit a substantially larger proportion of patients than most other biomarker-selected strategies. Until recently, KRAS was considered “undruggable” because of its molecular structure, but with the development of RAS(ON) inhibitors, allele-specific inhibitors and KRAS degraders, this paradigm has fundamentally changed [10,14,15]. This is further reflected by the late-phase clinical landscape identified in our analysis, where KRAS/RAS-directed therapies accounted for half of all phase III targeted trials (3/6).

Daraxonrasib represents an example of successful clinical translation within targeted therapy for PDAC. Encouraging phase I/II results in previously treated RAS-mutated pancreatic adenocarcinoma demonstrated a median OS of 14.5 months, establishing the rationale for phase III evaluation [32]. In a subsequent contextual update published after the registry dataset had been finalized, the benefit of daraxonrasib was demonstrated in the phase III RASolute 302 trial (NCT06625320), in which daraxonrasib significantly improved both OS (13.2 vs. 6.7 months) and PFS (7.2 vs. 3.6 months) compared with standard chemotherapy in the second-line setting. However, beyond the success of a single agent, these results provide the first clinically meaningful evidence that direct inhibition of oncogenic RAS signaling can improve survival in PDAC, representing a major milestone in precision oncology. Daraxonrasib is also being evaluated in the adjuvant phase III RASolute 304 trial (NCT07252232), suggesting that biomarker-derived targeted therapy could potentially be used in an earlier setting [33]. While the theoretical potential of KRAS targeting could also be useful in neoadjuvant treatment, currently, there are no late-stage trials in such a setting. Although KRAS-directed therapy may also have potential in the neoadjuvant setting, no late-phase neoadjuvant trials were identified.

Setidegrasib represents a complementary allele-specific strategy targeting KRAS G12D, the most common KRAS mutation in PDAC. Unlike daraxonrasib, which inhibits active RAS signaling across multiple RAS variants, setidegrasib is designed to selectively degrade the mutant KRAS G12D protein. Phase I studies demonstrated manageable safety together with preliminary antitumor activity in KRAS G12D-mutant solid tumors, including PDAC, supporting its progression into phase III evaluation in combination with mFOLFIRINOX or NALIRIFOX (NCT07409272) [34,35]. Collectively, these findings indicate that KRAS/RAS-directed therapies have progressed further than any other biomarker-selected strategy identified in our analysis. Their successful transition into phase III development suggests the potential of KRAS inhibition to become the first broadly applicable precision oncology strategy incorporated into routine PDAC treatment. In contrast, no other biomarker-selected approach has yet achieved a comparable level of late-phase clinical maturity.

HRD-associated therapies represent one of the few biomarker-selected strategies currently incorporated into treatment guidelines. This is largely based on the phase III POLO trial, in which maintenance olaparib significantly prolonged PFS (7.4 vs. 3.8 months) in patients with metastatic germline BRCA-mutated PDAC whose disease had not progressed following first-line platinum-based chemotherapy, although no OS benefit was demonstrated [36]. These findings illustrate both the potential and the limitations of precision oncology in HRD-associated PDAC. While PARP inhibition clearly benefits a carefully selected subgroup of patients, the absence of an OS benefit suggests that further optimization of treatment strategies remains necessary. Consequently, PARP inhibition currently remains relevant only for a small molecularly defined subgroup of patients, while platinum sensitivity continues to represent the most clinically validated therapeutic phenotype within HRD-associated PDAC. Consistent with this, all three late-phase HRD trials identified in our analysis (NCT06783140, NCT06115499, NCT06095141) evaluate platinum-based treatment strategies in molecularly selected BRCA1/2- or PALB2-associated populations rather than novel PARP-directed approaches [3,37,38,39].

Another notable example of biomarker-guided targeted therapy that has progressed to phase III evaluation is the PAN-PDAC trial (NCT06998940), which evaluates panitumumab in combination with chemotherapy in patients with KRAS wild-type PDAC [40]. Rather than introducing a new therapeutic target, this trial revisits an established therapeutic modality, EGFR inhibition, using a biomarker-selected approach, unlike previous studies that evaluated EGFR-targeted therapies in unselected PDAC populations, like the phase III SWOG S0205 trial of cetuximab plus gemcitabine, which failed to demonstrate meaningful improvements in survival [41]. If successful, the PAN-PDAC trial could redefine the role of EGFR inhibition in PDAC and establish a new targeted treatment option for the small subgroup of patients with KRAS wild-type disease.

Among the remaining targeted approaches, only quemliclustat is evaluated in a phase III trial (PRISM-1 (NCT06608927)), where it is combined with gemcitabine and nab-paclitaxel versus chemotherapy alone in treatment-naïve metastaticPDAC [42]. Unlike KRAS or HRD-directed therapies, this strategy does not target a tumor-specific genetic alteration but rather aims to overcome one of the key mechanisms of immune resistance-the tumor microenvironment. CD73 promotes the generation of immunosuppressive adenosine, thereby suppressing antitumor immune responses and potentially limiting the effectiveness of systemic therapy. In the phase Ib ARC-8 study, the addition of quemliclustat, a CD73 inhibitor, to gemcitabine and nab-paclitaxel demonstrated promising antitumor activity and a median OS of 15.7 months, providing a rationale for phase III development [43]. If confirmed in PRISM-1, these findings could establish CD73 inhibition as the first clinically relevant strategy targeting the immunosuppressive tumor microenvironment in PDAC.

Many other evaluated biomarker-selected approaches are directed against substantially smaller molecular subgroups and require prospective biomarker identification or are based on more recently recognized biological mechanisms, factors that inevitably prolong clinical development. Consequently, other molecular targets, including Claudin 18.2, MTAP-associated vulnerabilities, mesothelin, and several emerging precision oncology strategies, remain almost exclusively represented by early-phase clinical trials. Nevertheless, their continued expansion highlights the increasing diversification of precision oncology in PDAC.

Despite representing one of the largest therapeutic categories in our analysis, only a small number of immunotherapy studies were investigated in a late-phase trial. This is consistent with the unique biology of PDAC, where a dense desmoplastic stroma, a highly immunosuppressive tumor microenvironment and a relatively low tumor mutational burden collectively limit the effectiveness of immune checkpoint inhibition [7,8,44,45,46]. Consequently, clinical benefit from immunotherapy remains largely restricted to the small subgroup of patients with mismatch repair-deficient or microsatellite instability-high tumors, for whom current ESMO and NCCN guidelines recommend immune checkpoint inhibitors [2,3]. Rather than evaluating immune checkpoint inhibition as a standalone strategy, all late-phase immunotherapy-containing trials identified in our analysis investigate either combination with other treatment modalities or biomarker-guided patient selection. The phase III WGOG-PAN006/ICSBR-2 trial (NCT07336953) combines stereotactic body radiotherapy, serplulimab and gemcitabine plus nab-paclitaxel following encouraging phase II results demonstrating a promising 6-month PFS [47,48]. Rather than validating PD-1 blockade alone, this study is based on the concept that chemotherapy and radiotherapy may remodel the tumor microenvironment, thereby enhancing antitumor immune responses and improving the efficacy of immune checkpoint inhibition. Similarly, phase III trial NCT04969731 evaluates adjuvant Immuncell-LC in combination with gemcitabine following curative resection [49]. Unlike checkpoint inhibition, this approach aims to augment antitumor immunity through adoptive immune-cell therapy. Its progression into phase III evaluation was supported by encouraging clinical experience with cytokine-induced killer (CIK) cell therapies, which demonstrated a favorable safety profile together with preliminary efficacy signals in solid tumors, particularly hepatocellular carcinoma, providing the rationale for evaluation in PDAC [50]. A different concept is explored in the phase III trial NCT05482516, which evaluates novel immunotherapeutic strategies in patients with ctDNA-positive gastrointestinal cancers, including PDAC. Rather than introducing a new immunotherapeutic agent, this study builds upon accumulating evidence that ctDNA can identify molecular residual disease and patients at particularly high risk of recurrence [51]. Furthermore, observational studies have shown that ctDNA clearance during treatment is associated with improved survival [52]. This trial therefore reflects a shift towards biomarker-guided immunotherapy, where treatment is directed to patients most likely to benefit from it, rather than being administered to unselected populations.

### 4.4. Non-Systemic Treatment and Multimodal Treatment Approach

While systemic therapies dominate the current clinical trial landscape, our analysis demonstrates that several non-systemic treatment strategies have also progressed into phase III evaluation. Unlike many early-phase investigational approaches that primarily assess biological activity and safety, these studies focus on optimizing existing treatment pathways through improved integration of surgery, radiotherapy, local ablative techniques, and supportive care. This suggests that future improvements in PDAC outcomes are likely to arise not only from the development of novel drugs but also from combining multimodal treatment strategies.

Current ESMO and NCCN guidelines reserve radiotherapy for selected patients with borderline resectable or locally advanced PDAC, primarily following induction chemotherapy, where its role is to improve local disease control rather than overall survival [2,3]. This recommendation is largely based on the 2016 phase III LAP07 trial, which demonstrated improved local control without an overall survival benefit [53]. This leaves a gap to be filled by modern radiotherapy techniques and combinations which have since been developed. Consistent with this, four radiotherapy-based phase III trials were identified in our analysis, each addressing a different limitation of previous radiotherapy approaches. The NRG-GI011/LAP100 trial (NCT06958328) evaluates dose-escalated radiotherapy following induction chemotherapy in patients with locally advanced unresectable pancreatic adenocarcinoma, investigating whether modern dose-escalated techniques can improve clinical outcomes beyond those achieved with conventional radiation approaches [54].

The EXPAND trial (NCT06593431) evaluates metastasis-directed consolidative radiation added to systemic therapy, following the results of phase II EXTEND trial, where the addition of metastasis-directed therapy to standard systemic treatment significantly prolonged PFS (10.3 vs. 2.5 months) without increasing grade ≥ 3 treatment-related toxicity [55,56]. The third phase III radiotherapy-based trial (NCT06250972) evaluates IMRT following chemotherapy in patients with CA19-9-elevated locally advanced PDAC [57]. Unlike the other mentioned radiotherapy-containing trials, this trial was not preceded by a positive phase II study but was designed based on accumulating evidence that persistently elevated CA19-9 identifies patients with poor prognosis, following data from retrospective studies [58]. Together, these phase III trials show that radiotherapy research in PDAC has moved away from conventional chemoradiotherapy alone. The current focus is on dose escalation, stereotactic or image-guided treatment, oligometastatic consolidation and biomarker-based patient selection. This suggests that the future role of radiotherapy, if these trials succeed, will likely be in carefully selected clinical scenarios rather than as a broadly applied treatment for all patients with locally advanced or metastatic disease.

The three phase III surgery-related trials identified in our analysis focus on different aspects of surgical management. Two trials—PREOPANC-3 (NCT04927780) and NCT07081360—evaluate the optimal integration of surgery with systemic therapy. PREOPANC-3 compares perioperative with adjuvant mFOLFIRINOX in patients with resectable PDAC, while NCT07081360 compares neoadjuvant therapy with upfront surgery in patients with resectable left-sided PDAC [59,60].

In contrast, the Glubran2 trial (NCT07155525) focuses on perioperative surgical quality rather than treatment sequencing. It evaluates reinforcement of the pancreaticojejunostomy using tissue adhesive to reduce postoperative pancreatic fistula, one of the most frequent and clinically relevant complications following pancreaticoduodenectomy. By aiming to reduce postoperative morbidity and facilitate recovery, this trial highlights the importance of optimizing surgical outcomes to enable timely delivery of planned adjuvant treatment [61].

Among local therapies, irreversible electroporation (IRE; NanoKnife) was the only local intervention identified in a phase III trial (NCT03899636). Unlike thermal ablative techniques, irreversible electroporation (IRE) uses high-voltage electrical pulses to induce tumor cell death while preserving adjacent vessels and bile ducts, making it particularly suitable for locally advanced PDACs that encase major vascular structures [62]. Its progression into late-phase evaluation was supported by the phase II PANFIRE-2 study, which demonstrated that percutaneous IRE was feasible, had an acceptable safety profile, and achieved a median OS of approximately 17 months following IRE in patients with locally advanced PDAC after induction chemotherapy [63]. However, because these studies were non-randomized and included highly selected patients, it remains uncertain whether the observed survival benefit reflects the effect of IRE itself or favorable tumor biology.

Supportive-care studies represented a relatively small proportion of identified trials, although several were late-phase, with two being in phase III evaluation. The phase III Kinisoquin trial (NCT06861088) evaluates thromboprophylaxis in patients with advanced PDAC receiving systemic therapy. Given that PDAC carries one of the highest risks of cancer-associated venous thromboembolism, this trial addresses an important supportive care need that directly affects morbidity, mortality, and continuation of anticancer treatment [64,65]. The other phase III supportive care study (NCT06018883) evaluates low-dose ascorbate as an addition to gemcitabine and nab-paclitaxel with the aim of reducing chemotherapy-related anemia and improving treatment tolerability [66]. This is based on the established role of vitamin C in enhancing iron absorption together with preliminary clinical observations suggesting improvements in hemoglobin levels, chemotherapy tolerability, and quality of life [67]. Since these findings have not yet been confirmed in randomized trials, this trial has the potential to provide the first clinically relevant evidence for the role of vitamin C in the supportive management of patients with PDAC. A notable phase II/III supportive care trial is focused on ponsegromab (NCT06989437), a growth-differentiation factor 15 (GDF-15)-targeting cancer cachexia medication. It is based on encouraging phase II results demonstrating improvements in body weight, physical activity, and patient-reported outcomes [68,69]. This trial is particularly relevant because cachexia affects approximately 80% of patients with pancreatic cancer and is associated with poorer survival and quality of life [70].

Although no imaging trial was evaluated in a phase III trial, they address an unmet need in PDAC management. Conventional imaging has limited ability to accurately distinguish tumor tissue from treatment-related fibrosis and to detect small-volume metastatic disease [71]. Consequently, current research is focused on developing imaging techniques capable of more accurately characterising tumour biology and treatment response. This is reflected in two phase II/III trials; the PERISCOPE trial (NCT07219238) evaluates the diagnostic performance of the novel ^68Ga-GEH300079 PET/CT tracer, while NCT06782412 investigates [^18F]AlF-FAPI-74 PET imaging [72,73].

## 5. Strengths and Limitations

Our analysis has several strengths. To our knowledge, it represents one of the most comprehensive contemporary assessments of the active PDAC clinical trial landscape, encompassing 355 interventional studies registered between 2021 and 2026. In addition to characterizing therapeutic categories, the analysis evaluated disease-stage distribution, endpoint selection, funding patterns, and the characteristics of phase II/III and phase III studies. Furthermore, it provides an updated overview of emerging therapeutic approaches, including KRAS-directed therapies, Claudin 18.2-targeted therapies, MTAP-associated strategies, CD73 inhibition, and contemporary biomarker-selected treatment approaches that were not represented or were only minimally represented in earlier analyses.

This study also has several limitations. First, ClinicalTrials.gov was used as the primary data source. Although it is the largest international registry and captures many multicenter studies, trials registered exclusively in other regional databases may have been missed. Second, the analysis relied on registry-reported information, which may be incomplete or inconsistently updated, particularly for mechanisms of action, biomarker criteria, disease setting, and endpoint definitions. Third, completed, terminated, withdrawn, and suspended trials were excluded because the objective was to characterize the active investigational pipeline; therefore, the analysis does not capture the full history of failed or completed PDAC development. Fourth, therapeutic categories were not mutually exclusive, reflecting the reality that many modern trials combine chemotherapy, targeted therapy, immunotherapy, radiotherapy, surgery, or local interventions. Finally, efficacy outcomes were not systematically extracted from all studies; published phase I/II or phase III data were discussed only to contextualize major late-phase programs and should not be interpreted as a formal comparative efficacy analysis.

## 6. Future Directions and Conclusions

The contemporary PDAC clinical trial landscape reflects a clear transition from empirical treatment strategies towards increasingly biomarker-informed and multidisciplinary approaches. Although chemotherapy remains the therapeutic backbone and continues to dominate late-phase clinical development, the landscape is shifting towards targeted therapies, immunotherapy combinations, radiotherapy, and other treatment modalities Among biomarker-selected approaches, KRAS/RAS-directed therapies have demonstrated the greatest clinical maturity and currently represent the most promising strategy for broad implementation of precision oncology in PDAC. At the same time, continued development of HRD-associated, Claudin 18.2-targeted, MTAP-associated, EGFR-directed, and CD73-targeted therapies highlights ongoing efforts to expand the proportion of patients eligible for molecularly guided treatment. Beyond systemic therapies, advances in radiotherapy, surgery, local ablative techniques, supportive care, and molecular imaging further emphasize the importance of multidisciplinary optimization across the entire treatment pathway. Collectively, these findings suggest that future improvements in PDAC outcomes are likely to arise from the coordinated integration of precision oncology, optimized chemotherapy, and multidisciplinary treatment strategies into personalized care pathways.

## Data Availability

No new data were created or analyzed in this study. Data sharing is not applicable to this article.

## Supplementary Materials

The following supporting information can be downloaded at: https://www.mdpi.com/article/10.3390/ph19091371/s1, Supplementary Table S1: Characteristics of active clinical trials in pancreatic ductal adenocarcinoma included in the analysis.

## Abbreviations

The following abbreviations are used in this manuscript:

68Ga	Gallium-68
AE	Adverse Event
BRCA1	Breast Cancer Gene 1
BRCA2	Breast Cancer Gene 2
CA19-9	Carbohydrate Antigen 19-9
CAR	Chimeric Antigen Receptor
CAR-NK	Chimeric Antigen Receptor Natural Killer
CAR-T	Chimeric Antigen Receptor T cell
CD73	Cluster of Differentiation 73
CDH17	Cadherin-17
CEACAM5	Carcinoembryonic Antigen-Related Cell Adhesion Molecule 5
CIK	Cytokine-Induced Killer
ctDNA	Circulating Tumor DNA
DCR	Disease Control Rate
DLT	Dose-Limiting Toxicity
DoR	Duration of Response
EGFR	Epidermal Growth Factor Receptor
ESMO	European Society for Medical Oncology
FAK	Focal Adhesion Kinase
FGFR	Fibroblast Growth Factor Receptor
FOLFIRINOX	Leucovorin, Fluorouracil, Irinotecan and Oxaliplatin
GDF-15	Growth Differentiation Factor 15
HRD	Homologous Recombination Deficiency
ICI	Immune Checkpoint Inhibitor
IMRT	Intensity-Modulated Radiation Therapy
IRE	Irreversible Electroporation
KRAS	Kirsten Rat Sarcoma Viral Oncogene Homolog
MAPK	Mitogen-Activated Protein Kinase
MAT2A	Methionine Adenosyltransferase 2A
mFOLFIRINOX	Modified FOLFIRINOX
MRI	Magnetic Resonance Imaging
MSI-H	Microsatellite Instability-High
MTAP	Methylthioadenosine Phosphorylase
MTD	Maximum Tolerated Dose
mRNA	Messenger RNA
NALIRIFOX	Liposomal Irinotecan, Fluorouracil, Leucovorin and Oxaliplatin
NCCN	National Comprehensive Cancer Network
NIH	National Institutes of Health
ORR	Objective Response Rate
OS	Overall Survival
PALB2	Partner and Localizer of BRCA2
PARP	Poly(ADP-ribose) Polymerase
PD-1	Programmed Cell Death Protein 1
PDAC	Pancreatic Ductal Adenocarcinoma
PET	Positron Emission Tomography
PFS	Progression-Free Survival
PRMT5	Protein Arginine Methyltransferase 5
RP2D	Recommended Phase II Dose
SAE	Serious Adverse Event
SBRT	Stereotactic Body Radiation Therapy
siRNA	Small Interfering RNA
TCR-T	T-cell Receptor-Engineered T cell
TEAE	Treatment-Emergent Adverse Event
TGF-β2	Transforming Growth Factor Beta 2
TIL	Tumour-Infiltrating Lymphocyte
TROP2	Trophoblast Cell Surface Antigen 2
TTR	Time to Response
VEGF	Vascular Endothelial Growth Factor
